# Experiences and Perceptions of Telehealth Visits in Diabetes Care During and After the COVID-19 Pandemic Among Adults With Type 2 Diabetes and Their Providers: Qualitative Study

**DOI:** 10.2196/44283

**Published:** 2023-07-18

**Authors:** Chun-An Sun, Zachary Shenk, Susan Renda, Nisa Maruthur, Stanley Zheng, Nancy Perrin, Scott Levin, Hae-Ra Han

**Affiliations:** 1 Johns Hopkins School of Nursing Baltimore, MD United States; 2 Division of General Internal Medicine Johns Hopkins School of Medicine Baltimore, MD United States; 3 Department of Epidemiology Johns Hopkins Bloomberg School of Public Health Baltimore, MD United States; 4 Department of Health Policy and Management Johns Hopkins Bloomberg School of Public Health Baltimore, MD United States; 5 Department of Emergency Medicine Johns Hopkins School of Medicine Baltimore, MD United States; 6 Center for Data Science in Emergency Medicine Johns Hopkins School of Medicine Baltimore, MD United States; 7 Department of Health, Behavior, and Society Johns Hopkins Bloomberg School of Public Health Baltimore, MD United States

**Keywords:** telehealth, type 2 diabetes mellitus, adults, user experience, care continuity

## Abstract

**Background:**

Since the COVID-19 pandemic, telehealth has been widely adopted in outpatient settings in the United States. Although telehealth visits are publicly accepted in different settings, little is known about the situation after the wide adoption of telehealth from the perspectives of adults with type 2 diabetes mellitus (T2D) and their providers.

**Objective:**

This study aims to identify barriers and facilitators of maintaining continuity of care using telehealth for patients with T2D in a diabetes specialty clinic.

**Methods:**

As the second phase of a multimethod study to understand missed appointments among adults with T2D, we conducted semistructured, individual, in-depth phone or Zoom interviews with 23 adults with T2D (14/23, 61% women; mean age 55.1, SD 14.4, range 35-77 years) and 10 providers from diabetes clinics in a tertiary academic medical center in Maryland. Interviews were audio-recorded, transcribed, and analyzed using thematic content analysis by the research team.

**Results:**

Adults with T2D and their providers generally reported positive experiences with telehealth visits for diabetes care with some technical challenges resulting in the need for in-person visits. We identified the following 3 themes: (1) “perceived benefits of telehealth visits,” such as convenience, time and financial efficiencies, and independence from caregivers, benefits shared by both patients and providers; (2) “perceived technological challenges of telehealth visits,” such as disparities in digital health literacy, frustration caused by unstable internet connection, and difficulty sharing glucose data, challenges shared by both patients and providers; and (3) “impact of telehealth visits on the quality of diabetes care,” including lack of diabetes quality measures and needs and preferences for in-person visits, shared mainly from providers’ perspectives with some patient input.

**Conclusions:**

Telehealth is generally received positively in diabetes care with some persistent challenges that might compromise the quality of diabetes care. Telehealth technology and glucose data platforms must incorporate user experience and user-centered design to optimize telehealth use in diabetes care. Clinical practices need to consider new workflows for telehealth visits to facilitate easier follow-up scheduling and lab completion. Future research to investigate the ideal balance between in-person and telehealth visits in diabetes care is warranted to enhance the quality of diabetes care and to optimize diabetes outcomes. Policy flexibilities should also be considered to broaden access to diabetes care for all patients with T2D.

## Introduction

It is estimated that there are more than 37 million people in the United States with diabetes mellitus, and 90% to 95% of those cases are type 2 diabetes mellitus (T2D) [1]. Working with health care professionals is essential for diabetes management [2,3], but 12% to 36% of people with diabetes did not attend their regular medical appointments before the COVID-19 pandemic [4,5].

Telehealth, the use of telecommunication technologies to provide health care remotely [6], has been widely adopted in the United States in outpatient settings since the COVID-19 pandemic began in 2020, to mitigate the spread of COVID-19 [7]. Telehealth visits reduced people’s travel time and expenses, limiting COVID-19 exposure, and enabled clinicians to view a person’s lifestyle and environment [8-10]. Telehealth has been publicly accepted as a form of consultation and perceived positively by patients, caregivers, and providers in cancer, nephrology, and primary care settings [8-14]. The limitations cited by both patients and providers included technological challenges and the inability to perform a physical exam [8,10,12].

Specific to diabetes care, the transformation from in-person to telehealth visits has the potential to democratize routine diabetes care provision, such as providing care to those with limited transportation who otherwise could not be at the clinic regularly [15,16]. A previous study found that telehealth visits reduced the odds of missed appointments by more than 50% among adults with T2D, compared with in-person visits [17]. Telehealth visits also addressed some barriers (eg, better access to appointments, shorter travel time) of in-person visits in a group of veterans with T2D living in rural areas before COVID-19 [18]. Since the COVID-19 outbreak, many people with T2D have tried telehealth for the first time, with positive perceptions because no in-person care was provided during stay-at-home orders and insurance coverage expanded [19,20]. Promisingly, the reduction of in-person visits and increase of telehealth visits during the COVID-19 pandemic did not result in compromised glycemic control among adults with T2D with commercial or Medicare Advantage health plans [21].

However, some endocrinologists believed telehealth visits were more helpful for people with type 1 diabetes mellitus (T1D) compared with those with T2D because people with T1D are more closely monitored by continuous glucose monitoring (CGM) and insulin pumps [22]. Additionally, some patients with T2D and providers had concerns related to confidentiality and the quality of physical exams during a telehealth visit [20]. A systematic review of 20 articles on telehealth and telemonitoring in diabetes care found scant evidence examining the preferences and satisfaction of people with T2D in using telehealth [23]. Given that little is known regarding the impact of telehealth on T2D care from both patients’ and providers’ perspectives after the wide adoption of telehealth visits during the pandemic, the purpose of this study was to identify barriers and facilitators of maintaining continuity of care using telehealth for patients with T2D in a diabetes specialty clinic.

## Methods

### Study Setting

This qualitative narrative study took place in the Johns Hopkins Diabetes Center, a multidisciplinary team (physicians, endocrinology fellows, nurse practitioners [NPs], registered dietitians, and certified diabetes care and education specialists) providing comprehensive diabetes care across multiple locations in the greater Baltimore area affiliated with a tertiary academic medical center in Maryland. There were no telehealth (video or phone) visits before the COVID-19 pandemic. During COVID-19, appointments at the Johns Hopkins Diabetes Center were transferred to telehealth visits on March 23, 2020, after the expansion of coverage by the Centers for Medicare & Medicaid Services [24]. The clinics started scheduling both in-person and telehealth visits based on patients’ preferences and their COVID-19 risk around September 2020. Around the time of the interview, the clinicians would have at least one session (4-hour block) for telehealth visits per week.

In normal circumstances, the telehealth visits at Johns Hopkins are through MyChart (Webex by Cisco). A medical assistant usually calls a day before the appointment to verify patients’ physical locations to meet individual state regulations. On the day of the appointment, vital signs and medical history are verified again through a phone call after a patient completes the electronic check-in through MyChart. Before the scheduled appointment time, the link to “start your video visit” appears to initiate the visit. The link for the video visit can be sent via text message if a patient does not use MyChart. A simple phone call may be used if the patient does not have devices with video capabilities [25].

### Recruitment and Study Participants

The findings for this manuscript were the second part of a multimethod study focusing on missed appointments among adults with T2D. In short, the first part of the study used electronic health records (EHRs) to examine if predictors of missed appointments differ (1) between pre-COVID-19 (January 2019 to March 2020) and COVID-19 (March 2020 to December 2020) periods and (2) by health care delivery modes (in-person or telehealth visits) during COVID-19 among adults with T2D; more information is described elsewhere [17].

Based on the results of the first phase, the eligibility criteria for this qualitative study included a diagnosis of T2D, age greater than 18 years, residence in the state of Maryland, and at least one appointment with either a physician or an NP marked as a no-show in the EHR in the past year at the time of recruitment (February 2022 to July 2022). Adults who met the inclusion criteria were identified from the EHR and then were contacted via emails, phone calls, or text messages. We used purposive sampling with maximum variation to include adults from different physicians and NPs with diverse characteristics (eg, age [<60 years old, 61-75 years old, >75 years old], sex [female, male], race/ethnicity [Black, White, other], health care delivery modes [in-person or telehealth visits]) based on our quantitative results [17].

Potential provider participants were invited through a study presentation by one of the authors (CAS) at the monthly diabetes center meeting. CAS then emailed the study information to all potential provider participants. All physicians and NPs working at the Johns Hopkins Diabetes Center were eligible to participate.

### Ethical Considerations

The Johns Hopkins Medicine Institutional Review Board (IRB; #IRB00231790) approved the study design and procedures. Verbal consent was obtained from each participant.

### Data Collection

The study team developed a semistructured interview guide to explore perceptions among people with T2D and their providers regarding the barriers and facilitators of keeping appointments for both in-person and telehealth visits, with a focus on interpersonal relationships. The initial interview guide drafted by CAS was based on the findings of our previous quantitative study on missed appointments during the COVID-19 pandemic [17] and previous literature on user perceptions of telehealth [18-20,22]. Interview questions were revised and discussed among team members after the first 3 interviews with patients and providers. Example questions and probes to patient participants included the following: “Can you describe your experience doing a telehealth/video visit with your diabetes providers?” “What do you like the most and why?” Example questions and probes to provider participants included the following: “What is your experience of telehealth visits?” “How do you like it?” “What are the differences between providing care via telehealth or in-person visits?” The final qualitative interview guides are included in Multimedia Appendix 1.

One-on-one 30-minute to 45-minute phone or Zoom interviews were conducted by 2 trained interviewers (CAS and ZS) from February 2022 to July 2022 until thematic saturation was reached. In total, we contacted 52 eligible people with T2D; 28 refused (ie, not interested in the study topics, no times to be interviewed, no replies after 3 calls or text messages), and 24 agreed to participate. Of those participants agreeing to participate, 23 completed the interview, and 1 participant was excluded due to a mislabeled T2D diagnosis on the EHR. Among 12 eligible providers, 10 (8 physicians and 2 NPs) agreed to participate and completed the interviews. All phone interviews were audio-recorded; all Zoom interviews were recorded using Zoom’s recording features. Only audio recordings were saved and sent to a professional transcription vendor approved by the Johns Hopkins IRB for verbatim transcription. After verifying the accuracy and removing identifiable information, transcripts were imported into f4analyse for coding and analysis.

### Data Analysis

The analysis began after the transcription of the first interview and continued throughout data collection. We conducted a thematic content analysis of the interview transcripts, a common method of analysis when coding categories are derived directly from data [26,27]. Each transcript was read and coded independently by at least 2 of the 3 coders (CAS, ZS, and SZ). The study team identified codes for patients and providers separately. Any discrepancies in the codes were resolved through discussions referring to the transcripts until at least 2 study members reached a consensus to ensure coding consistency. Each coder then grouped similar codes into emerging themes by patients and providers. The study team discussed all emerging themes and finalized themes and subthemes. During the ongoing discussions, the study team realized some themes were similar across patient and provider participants and, thus, decided to further merge the themes as one.

### Rigor

Reflexivity was achieved through discussions between interviewers and written notes after the interview. Trustworthiness was maintained through team discussions and audit trail documentation; all documentation was kept using Microsoft Word. The transferability of the study was enhanced by providing a thorough description of the study method, study setting, and our processes of data collection [28].

## Results

### Participant Characteristics

#### Patient Characteristics

Details of characteristics of persons with T2D included in this study can be found in Table 1. Among 23 participants, >50% (14/23, 61%) were female, with an average age of 55.1 (SD 14.4) years. The majority of participants were Black, and all were non-Hispanic. Of the adult participants, 87% (20/23) had an active patient portal account, although 1 person had never logged in and 1 person had logged in more than a year ago at the time of the interview.

**Table 1:** Characteristics of persons with types 2 diabetes mellitus (T2D) at the time of the interview (n=23).

Characteristics		Results
Age (years), mean (SD; range)		55.1 (14.4; 35-77)
**Sex, n (%)**		
	Female	14 (61)
**Race, n (%)**		
	Black	12 (52)
	White	10 (44)
	Other	1 (4)
**Insurance, n (%)**		
	Medicare	12 (52)
	Medicaid	7 (30)
	Commercial	4 (17)
**Diabetes medications, n (%)**		
	Any insulin	16 (70)
**Continuous glucose monitoring, n (%)**		
	Ever used	8 (35)
**Patient portal account, n (%)**		
	Activated	20 (87)
**Time of care initiation, n (%)**		
	Before March 2020	15 (65)
**Health care delivery experience, n (%)**		
	In-person visits only	4 (17)
	Both in-person and telehealth visits	19 (83)

#### Provider Characteristics

Among 10 provider participants (8 physicians, 2 NPs), there were 7 female and 3 male providers, with an average service time at the institution of 7.2 (SD 4.54, range 1-14) years.

### Qualitative Findings

#### Overview

We identified 3 themes with 13 subthemes addressing patients’ and providers’ perspectives on telehealth visits in T2D care (see Table 2). Both patients and providers identified multiple benefits of telehealth visits in diabetes care but with certain technical challenges. Those challenges in telehealth visits resulted in negative clinical implications and called for the need for at least one annual in-person visit in diabetes care.

In the following sections, we first describe the first theme, “perceived benefits of telehealth visits,” shared by both patients and providers, followed by the benefits noted by providers only. We then describe the second theme, “perceived technological challenges of telehealth visits,” shared by both patients and providers. Finally, we describe the third theme, “impact of telehealth visits on the quality of diabetes care,” as perceived by both patients and providers, though mainly from providers’ perspectives with some patient input. Direct quotes are presented in the following sections.

**Table 2:** Themes and subthemes from patients' and providers' perspectives.

Themes		Provided quotes	
		Patients	Providers
**Theme 1. Perceived benefits of telehealth visits in diabetes care**			
	Diabetes care “in the comfort of your home” at a time of one’s convenience	Yes	Yes
	Saving money and time due to no need for transportation	Yes	Yes
	Relief from relying on one’s caregiver and less “juggling to get it into our schedule”	Yes	Yes
	Efficient visits, fewer delays, and more time with adults with T2D^a^	No	Yes
**Theme 2. Perceived challenges related to telehealth visits**			
	Disparities in digital health literacy and lack of devices as barriers to telehealth visits	Yes	Yes
	Frustration caused by unstable internet connection	Yes	Yes
	Phone visits not encouraged	No	Yes
	Difficulty sharing glucose data	Yes	Yes
**Theme 3. Impact of telehealth visits on the quality of diabetes care**			
	A double-edged sword for care continuity	No	Yes
	Perceived incomplete visits due to no diabetes quality measures	Yes	Yes
	Compromised care quality due to unavailability of glucose data and/or unpreparedness of their patients	No	Yes
	Needs for and preferences of in-person visits	Yes	Yes

^a^T2D: type 2 diabetes mellitus.

#### Theme 1. Perceived Benefits of Telehealth Visits in Diabetes Care

The majority of persons with T2D and provider participants described positive experiences with telehealth visits due to several benefits, mainly noting increased convenience and efficiency as compared with in-person visits. We identified the following 5 subthemes: (1) diabetes care “in the comfort of your home” at a time of one’s convenience; (2) saving money and time due to no need for transportation; (3) relief from relying on one’s caregiver and less “juggling to get it in our schedule”; (4) efficient visits, fewer delays, and more time with patients; and (5) a good fit for data-driven diabetes care.

Regarding the first subtheme (diabetes care “in the comfort of your home” at a time of one’s convenience), many adults with T2D specifically cited the convenience of being able to conduct telehealth visits from their homes or at a private location conducive to their schedule. Similarly, from providers’ perspectives, many providers acknowledged the added convenience of telehealth visits for their patients, citing reasons largely in alignment with what patient participants mentioned:

It's really convenient, I don't have to take off from work, I don't have to park. Based on the time of the day, any time prior to like 3 o'clock, the parking garage is pretty well filled up. So, it just really alleviates a lot of additional stress, and a lot of the times, I can either do it at work in a secluded area. I think it's just great, very convenient.

Adult09

I think the benefit of telehealth is patients who are not coming in because of either distance or they can't find the time to come in at least we have this as a resort to say, “Well, if you can take 20 minutes out of your workday even at work, we can at least still see you and try to do some management.” I think it's useful in that sense.

Provider09

Regarding the second subtheme (saving money and time due to no need for transportation), people with T2D who drive to in-person visits expressed contentment about not having to pay for parking or gas. Adults who take public transportation to attend in-person visits were satisfied with telehealth visits due to saving money and decreased wait times for both transportation as well as in the provider’s office. Many providers empathized with people’s economic and time constraints that often hindered in-person visit attendance. The following quotes from a person with T2D and a provider reflect the mutual understanding of the perks of being able to conduct diabetes care visits irrespective of their physical locations:

Yeah, I do like the video visits. I mean, because it saves money. You know, you don't have to worry about transportation to and from. You know, you could be in the comfort of your own home.

Adult18

I think it (video visit) is a fantastic way to extend care to people who don't have to drive into our clinic, don't have to park, don't have to waste probably an hour to 2 hours of their day.

Provider06

Regarding the third subtheme (relief from relying on one’s caregiver and less “juggling to get it into our schedule”), several people with T2D shared their emotional burden of having to rely on their caregivers (ie, family) to attend in-person visits for a variety of reasons. For them, sharing one vehicle meant that family members had to miss time from work or skip their own medical appointments to help people with T2D with transportation to in-person diabetes visits, or sometimes the adults with T2D would need to skip their visit in lieu of their caregivers. Hence, when telehealth visits were available, these adults with T2D felt content as they no longer needed to rely on others for in-person visits. This quote highlights the experience of 1 participant who had a right leg amputation:

To me, it’s convenient that you don't always have to go into the hospital because my fiancée works 9 to 5, which is most of the time that appointments are, so she doesn't have to miss time from work to take me. Normally I could drive, but now with this amputation, I can't do that myself. So, they help a lot, that everything can be done right through a video call, don't actually have to be there.

Adult10

A provider also acknowledged the barriers of in-person visits, saying:

The patients, I think, though, have more of a struggle (doing in-person visits) than we do because of transportation and coming in. If they’re older and they don’t drive, and yet they need to be driven there, that kind of thing.

Provider04

The fourth subtheme (efficient visits, fewer delays, and more time with adults with T2D) was unique to providers. Providers described how telehealth visits can be conducted more efficiently than in-person visits, thereby reducing delays in their daily schedule and allowing them to maximize their time spent with each patient. One provider participant contrasted their use of time during in-person and telehealth visits and the beneficial impact on their daily workflow:

The person has to get transportation to (the clinic), and then maybe if they have a vehicle, they have to park it. There’s the process of getting signed in, and my 1:00 patient who arrives a couple minutes after 1:00, and then the medical assistant is a little busy, may not get them in the room until 1:25, which can then set my schedule behind. Whereas on telemedicine, I'm running it, I can say to a patient, if we need to wrap-up, like “We only have 5 more minutes,” and I know I’ll have somebody coming into the queue, who will be ready to go ahead and have their appointment. It’s easier to structure the patient to the amount of time you have and also make use of that full amount of appointment time, because you immediately sign onto them, so there’s no delay at all.

Provider04

The fifth subtheme (good fit for data-driven diabetes care) was unique to providers. Most providers mentioned that diabetes care was suitable for telehealth visits as it focused more on the behavioral and cognitive perspectives. However, providers also specifically expressed the requirement of having home blood glucose data to provide optimal diabetes care and a treatment plan to their patients. This practice is particularly important in telehealth visits as most adults might not have an available glycated hemoglobin (HbA_1c_) measurement for the telehealth visits as compared with in-person visits, where a point-of-care HbA_1c_ is taken. Advances in diabetes technology allow most people with diabetes to share their blood glucose data before telehealth visits.

Diabetes care, half the time, I don’t even put my hands on a patient, other than to look at their toes and maybe recheck their blood pressure. It really is whatever they say, a cognitive specialty. If they (adults) can share their readings from home, it can be just as good and preferable from the patient’s perspective.

Provider02

#### Theme 2. Perceived Challenges Related to Telehealth Visits

Despite the aforementioned benefits, patient and provider participants all faced some challenges related to technology during telehealth visits. To complete a successful telehealth visit in diabetes care, people with T2D needed to have a digital device, know how to get online, navigate the patient portal to the nested telehealth platform, upload their glucose data via a cloud or patient portal, and have familiarity with manipulating the video camera and volume. Any disruption could happen during this process, which could result in a suboptimal experience. We identified 3 subthemes related to technological challenges from both adults and providers and 1 subtheme unique to providers.

Regarding the first subtheme (disparities in digital health literacy and lack of devices as barriers to telehealth visits), when navigating technology aspects of telehealth (eg, using a digital device, patient portal, telehealth platform, uploading glucose data) throughout COVID-19, some people with T2D were proficient from the beginning, while others required additional support from staff at the diabetes clinics or other family members. For other patient participants, the repeated practices over time made them more comfortable with telehealth-related technology:

I've got a desktop that we've had for some time, and my wife is a technical genius in the house, she helped me get it set up three times now, I'm an old veteran; I could do it solo now.

Adult04

However, 2 people with T2D specifically cited computer illiteracy as the reason for not utilizing the patient portal (Adult06, Adult18). In this case, they would rely on providers to contact them using alternate video platforms (eg, Doximity, FaceTime) without going through the patient portal. All providers acknowledged that the use of telehealth required digital health literacy and were prepared to use different ways to connect with their patients. A provider discussing the limitations of telehealth visits mentioned the following:

Not everybody has a computer at home, which is a problem. Or they don’t have iPhones, so, we can’t do a Facetime call with them. (...) Any of these technical issues mean that we can’t cover as much in that half an hour as I would if I saw them in person because you don’t have those limitations in person.

Provider10

Both people with T2D and providers acknowledged that the lack of digital devices was a barrier to telehealth visits. At the time of the interview, 3 adults did not have a digital device with a camera capacity (Adult02, Adult07, Adult15). Of these adults, 2 had only phone visits during the peak of COVID-19, while the other person had only in-person visits as he established care after in-person visits resumed. One person who only had experiences with phone visits stated the following:

No. I don't even know how to work that (video visit), uh-uh. I just have telephone calls. I don't know how to do none of that virtual stuff. (...) I just upgraded my phone, but I'm saying I still don't know how to work it real good.

Adult07

Regarding the second subtheme (frustration caused by unstable internet connection), once people with T2D and providers were connected, echoing in voices and delay in transmission due to unstable internet connection were other issues that undermined the quality of conversation and sometimes caused frustration for both parties involved. As most of the allotted time was spent on nonmedical issues (ie, trying to get connected with each other), people did not have enough time to ask questions, and providers were unable to properly deliver care. A provider provided the following quote when discussing the disadvantages of telehealth visits:

It really comes down to the connection and the person’s savvy with it. I had a lady, lives in Virginia, and she’s elderly, and a friend had to drive her over the line into Maryland. So, they were in the car trying to get a connection with me, and where they were, it wasn’t good connection. So, I’m seeing her frozen face, she sees my frozen face, we halfway hear each other, and finally it had to degrade into a phone conversation.

Provider04

A person with T2D discussing this frustration in her previous telehealth experience mentioned the following:

It (video visit) didn’t go through. We were talking and then it kept disconnecting. The call kept dropping, so we had to wind up just texting. So, 1 out of 5, I’d give it a 1.  (...) I will not give it (the video visit) a try.

Adult21

The third subtheme (phone visits not encouraged) was unique to providers. At the time of the interview, phone visits were not encouraged due to the complexity of the reimbursement and compliance issues. Although phone visits were the least preferred method for providers to connect with their patients, it was a necessary backup when connection issues or the other abovementioned technical issues persisted.

You can't bill for phone visits. (...) Or you can bill for it, but you won't get reimbursed for it. So, then why did I go to all that trouble? Then, I can just have my secretary set up a phone call, and I can just have a phone call, which will work easier, instead of doing this thing of getting onto EPIC—it's a nightmare. Not good.

Provider06

Regarding the fourth subtheme (difficulty sharing glucose data), both people with T2D and providers mentioned the potential obstacles of sharing glucose data in telehealth visits. Depending on the devices (ie, CGM or glucometer) a person uses, sharing glucose data can be either easy or very troublesome. Patient participants with a CGM generally reported a smooth and easy process for sharing data compared with those with a traditional glucometer. However, not everyone with T2D was eligible for insurance coverage for a CGM, which is expensive for a person paying out of pocket. Many adult participants using a glucometer reported sending handwritten documents ahead of the telehealth visits or reading their daily glucose data in the past few weeks aloud during the telehealth visits. A person with T2D with experience using both glucose monitoring systems shared his experience:

Before, it (sharing glucose data) was really easy. Now that I've got different insurance, it's not as easy as it was before, because before I had a CGM and it could just upload the information. So, I just uploaded the information to my dock . Now I'm back to pricking my finger (glucometer), I can't (upload my data), it (the glucometer) didn’t do the same. I've got to keep a record of it myself and share it with my doctors.

Adult04

Sometimes, if a person did not have their glucometer with them, a provider would rely on the person’s memories of their glucose trends to decide on the treatment plan. Either way, this process took extra time for providers to make sense of the glucose data (eg, time in range, average, trends) before a clinical judgment was made. Those practices specific to telehealth visits either increased the time needed by providers or limited the length of a visit to address adults’ questions and concerns:

I had to have them (during telehealth) grab their glucometer and scroll through the numbers and read to me what those were, but for a 20-minute visit, if they're doing that for more than 10 minutes, it really leaves us not much time to go over other things.

Provider09

#### Theme 3. Impact of Telehealth Visits on the Quality of Diabetes Care

The aforementioned benefits and challenges of telehealth had clinical implications for diabetes care. We identified 4 subthemes mainly from providers’ perspectives with support from patient input, including “a double-edged sword for care continuity,” “perceived incomplete visits due to no diabetes quality measures,” “compromised care quality due to unavailability of glucose data and/or unpreparedness of their patients,” and “needs for and preferences of in-person visits.”

The first subtheme (a double-edged sword for care continuity) was unique to providers. With all the needed information on hand, telehealth visits allowed providers to offer more frequent quality diabetes care to adults with T2D who traditionally could not attend in-person visits often due to geographic barriers or other transportation-related issues:

I think this model (telehealth setting) has some advantages in that way, I mean they can touch base more frequently, they might be shorter communications, but they're more frequent as opposed to spaced out longer in-person visits.

Provider07

Provider participants also noticed that telehealth visits decreased missed appointments, which increased care continuity. Adults with T2D scheduling a telehealth visit received at least one extra reminder because current regulatory requirements mandated the verification of a person’s location before the visit. Additionally, due to the unpredictable nature of technical issues in telehealth visits, providers were more likely to outreach to adults despite an initial absence on the telehealth platforms. Most of their patients were able to remotely engage immediately in telehealth visits when prompted by a provider’s phone or video call. A provider described missed appointments and provided the following quote:

I will say, with telemedicine, it’s easier, because you can just call them and say “Hey, you have an appointment, let’s just talk right now.” A lot of people will say “Okay, that’s fine.”

Provider03

On the other hand, the current telehealth workflow in the diabetes clinics requires adults with T2D to take the initiative to schedule their next appointment after a telehealth visit, instead of scheduling the next appointment at the front desk on the way out of the office after an in-person visit. Although it did not bother people with T2D in this study, a person described how her other health conditions delayed her scheduling the next appointment:

I know I need to schedule an appointment. In fact, I was gonna call this week, but I'm getting a medical procedure, so Monday I was seeing other health care providers, so probably next week, I'll call the scheduling line and set up something or try and do it through MyChart to set up (the next appointment).

Adult13

The delay in scheduling the next appointment could sometimes lead to discontinuity in diabetes care because there was no follow-up mechanism at the time of the interview by the clinics after each visit. A provider discussing the impact of telehealth in diabetes care described this phenomenon:

Often, I will say they fall through the cracks, because we don't have the staff to follow-up on every patient and see if they scheduled a follow-up, and so it's sort of like I tell them “Please schedule a follow-up,” and I put in the order so that they'll get a prompt in MyChart, but beyond that we're not following-up to see what happens, and so they may not schedule it themselves. 

Provider05

Regarding the second subtheme, (perceived incomplete visits due to no diabetes quality measures), both people with T2D and providers viewed being unable to complete a thorough physical evaluation as a major limitation of telehealth visits. The American Diabetes Association recommends each person with T2D undergo a physical exam (eg, foot exam) and biofeedback (eg, BMI, blood pressure, HbA_1c_, lipid panel, microalbumin) quarterly or annually [3]. Instead of getting their point-of-care HbA_1c_ or other biofeedback at their in-person visits (lab facilities are available in the same building for all diabetes clinics), the responsibility of completing the required lab work shifted to adults with T2D after a telehealth visit; they must remember to make an additional trip to a lab facility. Additionally, it is not feasible to assess diabetes-related complications and other physical exams via telehealth. A person weighing in on telehealth visits mentioned:

I mean, I think that’s (video visits) good, but I can’t come in getting my instant test, how “boom,” they give you the A1c. It was awesome. I liked that.

Adult22

Providers admitted the same limitation, adding this additional effort sometimes led to incomplete diabetes quality measures. Without the information, providers might not be able to provide timely treatment recommendations, which could ultimately compromise their patients’ health outcomes. When comparing in-person and telehealth visits, a provider provided the following quote:

We can’t do a point-of-care A1c at those visits, and those are problems because we care about all of those and it impacts our decision making . (...) We try to reach out to patients who haven’t had labs done in a while to try to get their labs before their (video) visit, but that’s challenging because the point-of-care A1c makes it very (easy)—it’s a 5-minute test result.

Provider10

The third subtheme (compromised care quality due to unavailability of glucose data and/or unpreparedness of their patients) was unique to providers. Although telehealth visits had the potential to enhance care continuity through proactive outreach from a provider, provider participants felt that sometimes their patient was distracted during a telehealth visit—they might be driving with an intermittent internet connection, walking down the street, or having other commitments—so it was difficult to assess their lifestyle management during that environment. In addition to the inattention, people with T2D might not have their glucose data ready to share, as mentioned previously. Therefore, provider participants sometimes felt that a telehealth visit could compromise the care quality. A provider discussing frustration in telehealth visits mentioned the following:

There are times when telehealth visits are not productive. It really, I think, depends on patient engagement. I certainly have had patients who are taking the call while they're driving, or they forgot that they had a visit with me. So, in some ways, people take the telehealth less seriously, in which case, it's a waste of their time and so they're not ready for the visit and so sometimes they don't have labs, no glucometer data (...).

Provider09

Regarding the fourth subtheme (needs and preferences for in-person visits), both patient and provider participants shared the need for in-person visits. Although people with T2D in this study generally perceived that the conversations and interpersonal relationships during both telehealth and in-person visits were similar, only 5 participants noted a preference for telehealth visits; more than one-half of the participants specifically noted a preference for in-person visits because of the challenges and clinical implications discussed above:

Personally, I like face-to-face with my doctors, a checkup to see how you’re doing, with diabetes care. (...) It’s not like I have any kind of feet issues or circulation issues, hopefully never, but it’s good to go through that kind of stuff (check my feet) and just have them check that stuff, and it’s hard to do that with a virtual visit.

Adult20

Similarly, provider participants also mentioned that adults with T2D should have an in-person visit at least once a year:

I do think that patients do have to be seen at least annually, in-person, for a comprehensive foot exam, and other parts of the exam that need to be done as well. (...) What I was finding with telemedicine is that a lot of time, though the visits were great and the recommended frequency would continue, often, the labs would lag behind. Patients wouldn’t feel comfortable going to get their labs done.

Provider08

## Discussion

### Principal Findings

This qualitative study outlined the perspectives of both providers and patients with T2D on the benefits and challenges of telehealth in diabetes care. Although people with T2D and their providers acknowledged the convenience and efficiency of telehealth visits for promoting care continuity in diabetes care, telehealth also had challenges that could compromise the quality of diabetes care.

Consistent with previous literature [18], both adults with T2D and providers in this study acknowledged that telehealth visits addressed the barriers of transportation and work commitments with in-person visits. Beyond these benefits, providers in this study generally viewed intermittent telehealth visits as appropriate for diabetes care in the setting of a stable internet connection and the absence of technical issues. A cross-sectional study using national data and census data found that neighborhood broadband internet subscription was highly associated with the use of telehealth [29]. To mitigate widening disparities in access to care via telehealth services, state and federal governments should progressively invest in affordable household broadband internet infrastructure [30] and programs aiming to increase digital health literacy for all [31].

Given health care systems’ rapid increase in telehealth capacities since the COVID-19 pandemic [32], it is key to address the digital divide to ensure health equity by examining individual digital health literacy and the usability of the telehealth platforms. In our study, many adults with T2D had problems navigating through their smart devices or patient portal due to limited digital health literacy, but they indicated a willingness to use telehealth services with additional support. Quality improvement efforts to evaluate the uptake of telehealth services and specific measures to bridge digital literacy gaps, particularly among populations with limited resources, should be undertaken. For example, clinical practices may implement validated satisfaction surveys to identify digital literacy shortfalls and inform the development of staff training to better support patients in navigating through the platform [33,34]. Additionally, telehealth or health information platforms should seek to simplify the navigation of their systems with end user experiences in mind (eg, fewer layers to get to the actual link for telehealth visits) [35]. Last, clinical practices should consider new workflows for telehealth visits to facilitate easier follow-up scheduling and lab completion that include the perspectives of adults with T2D [36].

Several temporary policy flexibilities broadened access to diabetes care during the COVID-19 pandemic [15,16], including the coverage of audio-only visits [37] and the suspension of geographic requirements for patients [38]. However, with those flexibilities being phased out [39,40], telehealth care will be more limited, particularly to underserved populations. Currently, people living in rural areas across state lines must be present in the same state as their clinics’ locations to access care, further burdening those with limited resources. Additionally, we found that audio-only visits in diabetes care became necessary when technical issues arose, even though phone visits were not encouraged at the time of the interview due to reimbursement and compliance issues. Eliminating audio-only visits disproportionately affects certain populations, such as racial minority populations, those with public insurance, and older adults [41,42]. To ensure equitable access to diabetes care, new legislation and licensure registration should provide more flexibility in telehealth delivery [38].

Our study revealed concerns about glucose data availability impacting the quality of diabetes care in telehealth visits. Of the patients in this study, 70% (16/23) used insulin at the time of their interview, and glucose monitoring is integral to guiding individualized treatment plans in this population [43]. Sharing data, particularly from a glucometer, has been troublesome in telehealth visits as it requires extra steps and additional technological familiarity for people with T2D. Most participants (15/23, 65%) used a glucometer (finger sticks) for their daily glucose monitoring at the time of the interview, and none of them uploaded the data to the suggested platform (ie, Glooko). To enhance the quality of diabetes care and minimize burden, user experience and user-centered design should be considered in redesigning glucose-sharing platforms to minimize challenges faced by adults with T2D [44]. CGM, which is increasing in use for T2D, could also provide a convenient way to share glucose data in telehealth visits [45], but coverage for people with T2D remains limited [46]. With the potential to reduce inequality in diabetes burden and relevant complications [1], future research is warranted to investigate the benefits of CGM among individuals with non-insulin-dependent T2D. Insurance policies should also consider expanding CGM coverage to people using any insulin or oral medications with a higher risk of hypoglycemia (ie, sulfonylureas) and adults with physical, cognitive, or emotional barriers to finger sticks [47]. Additionally, although data platforms such as Glooko have been developed to address interoperability, none of the platforms can synchronize with all the commercially available diabetes devices (glucometers or CGMs). Moreover, diabetes data are not currently integrated in EHRs. More discussion on interoperability, integration, and patient privacy should be undertaken to enhance diabetes care for both clinicians and patients [48].

Both patient and provider participants in this study acknowledged that telehealth visits promote care continuity because of convenience and efficiency, but both indicated the need for in-person visits in T2D care. Attending in-person visits allows people with T2D to check diabetes quality measures (ie, foot exam, BMI, blood pressure, HbA_1c_, lipid panel, microalbumin [3]) within the same trip. During the COVID-19 pandemic, diabetes-related HbA_1c_ and nephropathy monitoring declined and did not recover to the prepandemic volume in the primary care setting [49]. A gap in timing between HbA_1c_ measurements was also a risk factor for missed appointments in the diabetes-specific setting [17]. To ensure care continuity and promote better outcomes, future research is warranted to investigate the ideal balance between in-person and telehealth visits in diabetes care.

### Limitations

This study has a few limitations. All participants were from diabetes clinics within a large urban academic medical center in the mid-Atlantic region of the United States. Additionally, the study was designed to focus on missed appointments and interpersonal relationships, and thus, themes presented in this study might not apply to the people with T2D who do not miss appointments. Last, this study collected data from participants who spoke English and responded to our phone calls, text messages, or emails. Although we maximized variations in recruiting participants (eg, based on age, race, and lengths of provider-patient relationship), the themes derived may not apply to people lacking a working phone number or who do not speak English.

### Conclusion

In summary, telehealth implementation during the COVID-19 pandemic has expanded access to diabetes care. Adults with T2D and providers generally reported positive experiences with telehealth visits, although some definite technical challenges exist. To ensure equitable access to diabetes care, legislation should provide more flexibility regarding geographic boundaries and telehealth delivery modes (audio-only versus video-audio visits). Telehealth-related technology design also needs to consider user experience and user-centered design to optimize the use of telehealth; a person-oriented telehealth workflow has the potential to address concerns about the negative effects of telehealth visits on the quality of diabetes. Future research to investigate the ideal balance between in-person and telehealth visits in diabetes care is warranted to enhance the quality of diabetes care to optimize diabetes outcomes.

## Appendix

Multimedia Appendix 1 Interview guides.

## Abbreviations

CGM: continuous glucose monitoring
EHR: electronic health record
HbA_1c_: hemoglobin A_1c_
IRB: institutional review board
NP: nurse practitioner
T2D: type 2 diabetes mellitus
