# Supplementary material for: Experiences and Perceptions of Telehealth Visits in Diabetes Care During and After the COVID-19 Pandemic Among Adults With Type 2 Diabetes and Their Providers: Qualitative Study
Source: JMIR Diabetes. 2023 Jul 18;8:e44283. doi: 10.2196/44283 (PMC10394605; doi:10.2196/44283)
Supplement: Multimedia Appendix 1 [file diabetes_v8i1e44283_app1.docx]

## QUALITATIVE INTERVIEW GUIDE (PATIENT)

**Warm-Up (2 mins) & Theme 1: General interaction with the health system (8 mins)**

*How would you describe your health?*

1. What types of doctors have you been seeing?
   1. Primary care
   2. Other specialty other than diabetes doctors (endocrinology)?
2. How did you hear about those doctors? /What made you decide to go to those doctors?
   1. Recommendation? Insurance? Transportation / Parking / Location?
3. How easy was it for you to get an appointment? (as a new patient, a recurring patient)
4. How did you like your providers (primary care, specialty)?
5. Was it easy to get a hold of your provider if you have questions?
6. What do you like the most about the doctor/ the clinic?
7. What’s the most frustrating things when seeing this doctor/ going to this clinic?

**Theme 2: Diabetes Center (10 mins)**

*Now we are switching gear to talk more about your experience at the Johns Hopkins Diabetes Center.*

1. (if haven’t mentioned in theme 1) Tell me about how you found out your diagnosis of type 2 diabetes.
   1. When? Where?
   2. What kind of treatment have been on? Whom were you seen before DM center?
2. When did you start seeing doctors at Johns Hopkins Diabetes Center? / How long have you been seen at the Diabetes Center?
   1. How did you know about the Diabetes Center? (Referral?)
3. Whom have you been seeing at the diabetes center?
   1. Physician? Nurse Practitioner?
4. How often do you have an appointment?
5. How easy was it for you to get an appointment or to modify your appointment?
   1. As a new/ returning patient
      - How did you make an appointment? Calling? MyChart?
6. How easy was it for you to get to the diabetes center? (transportation)
   1. Parking, public transportation?
7. How was the wait time before you being seen?
8. How do you like your DM doctors?
9. What do you like the most about the diabetes clinic?
10. What’s the most frustrating things when coming to the DM center?
    - - In-person vs telehealth (video/phone visit)
      - Contents of the conversation (lifestyle, preference, household situation)
      - Trust in providers/ Being heard
11. How easy for you to get a hold of Dr._____/NP______?

**Theme 3: Provider-related (5 mins)**

1. You told me you’ve been seeing _______, __________, and __________ at DM center. Whom did you see first? How were you told if you were going to see different providers the first time?
2. How do you like them?
   1. What are the same/difference?
3. Do you have a preference seeing one or the other? Why?

**Theme 4: Telemedicine (5 mins)**

1. Have you tried telemedicine/ telehealth/ video visit with your DM doctors?
   1. Yes- how do you like it?
   2. No- why was that?
      - How did you seek care during COVID? (might say phone call; treat it as video visit)
2. How did you share your sugar data or other information with your providers for a video visit?
3. What do you like about the in-person / video visits?
4. What do you dislike about the in-person / video visits?
5. Time/schedule
6. Transportation
7. Technology difficulty
8. Communication with the provider
9. Privacy
10. Follow-up

**Theme 5: COVID related (5 mins)**

1. Since we talked about video visit and COVID, we wanted to know how has COVID impacted you?
   1. Was it easy to get the health care you need?
   2. How about diabetes management? Exercise, eating?
   3. How about financial wise, did you have to skip meals or skip appointment/medication?
      - If yes, have you discussed with any concerns with any of your providers? Primary care? DM doctors? Receiving any recourses?

**Theme 6: No-show specific (5 mins)**

1. Have you ever been not showing up for an appointment without calling to cancel or cancel online?
2. What happened?
3. What were the challenges you have in keeping your appointment?
   - - Which provider they are going to see?
     - Day/time of the appointment?
     - Wait time?
     - Transportation? (where they live; public transportation or driving?)
     - Other responsibilities (job, housework?)
     - Comorbidities (have multiple diseases and many appointments)
     - Economic issues (insurance program/high copay)
     - Do not trust the provider? Do not find the care is useful
   1. What are the things that may help you to keep an appointment? Or cancel in advance?

That is all my questions for you. Before I stop recording, do you have any other questions or things to add regarding your appointment/visits at DM center?

Thank you so much for your time.

##

## QUALITATIVE INTERVIEW GUIDE (PROVIDER)

**General experiences**

1. How did you decide to work in endocrinology/ diabetes?
2. Please tell me about your career at the Johns Hopkins Diabetes Center.
   1. How long have you worked here?
   2. How often do you take care of a patient with type 2 diabetes?
3. If you are to share what the diabetes center is, how would you describe it?
   1. Please describe your routine for an appointment.
      - What kind of questions will you ask?
      - What information will you gather?
        1. Social history?
      - What material will you share to the patient? (handout? A to-do list?)
   2. I heard Diabetes Center is using a team-approach. Can you elaborate on that?
      - How often does your patient see another provider?
      - When will you “refer” your patient to see another provider?
      - How did you introduce your colleague?

**In-person vs telehealth visits**

1. What do you see the difference of seeing your provider via video/phone or in-person?
   1. Comparing to in-person visits, how do you like providing care via telehealth visits?
      - How does your patient usually share their glucose data?
      - Other technology issues?
   2. What are the advantages/disadvantages of in-person visits?
   3. What are the advantages/disadvantages of telehealth visits?
   4. What barriers of appointment no-show have telehealth visits removed?
   5. What are the barriers of appointment no-show that telehealth visits cannot remove?

**Barriers and facilitators to appointment-keeping behavior**

1. We would like to learn more about how people decide showing up to their appointment or not. From your experience, what is the greatest challenge for people to keep their appointment?
